# Extrapedicular Infiltration Anesthesia as an Improved Method of Local Anesthesia for Unipedicular Percutaneous Vertebroplasty or Percutaneous Kyphoplasty

**DOI:** 10.1155/2016/5086414

**Published:** 2016-09-28

**Authors:** Liehua Liu, Shiming Cheng, Rui Lu, Qiang Zhou

**Affiliations:** ^1^Department of Orthopedics, No. 13 People's Hospital of Chongqing, Chongqing 400053, China; ^2^Orthopedics Center of PLA, Department of Orthopedics, Southwest Hospital, Third Military Medical University, Chongqing 400038, China; ^3^Department of Orthopedics, Chongqing Dongnan Hospital, Chongqing 401336, China; ^4^Department of Anesthesia, Southwest Hospital, Third Military Medical University, Chongqing 400038, China

## Abstract

*Aim. *This report introduces extrapedicular infiltration anesthesia as an improved method of local anesthesia for unipedicular percutaneous vertebroplasty or percutaneous kyphoplasty.* Method.* From March 2015 to March 2016, 44 patients (11 males and 33 females) with osteoporotic vertebral compression fractures with a mean age of 71.4 ± 8.8 years (range: 60 to 89) received percutaneous vertebroplasty or percutaneous kyphoplasty. 24 patients were managed with conventional local infiltration anesthesia (CLIA) and 20 patients with both CLIA and extrapedicular infiltration anesthesia (EPIA). Patients evaluated intraoperative pain by means of the visual analogue score and were monitored during the procedure for additional sedative analgesia needs and for adverse nerve root effects.* Results.* VAS of CLIA + EPIA and CLIA group was 2.5 ± 0.7 and 4.3 ± 1.0, respectively, and there was significant difference (*P* = 0.001). In CLIA group, 1 patient required additional sedative analgesia, but in CLIA + EPIA group, no patients required that. In the two groups, no adverse nerve root effects were noted.* Summary. *Extrapedicular infiltration anesthesia provided good local anesthetic effects without significant complications. This method deserves further consideration for use in unipedicular percutaneous vertebroplasty and percutaneous kyphoplasty.

## 1. Introduction

Since the first reports of percutaneous vertebroplasty procedures in 1987, percutaneous vertebroplasty (PVP) and percutaneous kyphoplasty (PKP) have been widely used to treat osteoporotic vertebral compression fractures, myeloma, and vertebral metastases. Advantages of the procedure include minimal surgical invasiveness, good pain relief, and rapid recovery [1–3].

Recent reports have claimed similar biomechanical stability and clinical efficacy of unipedicular PVP (PKP) as compared to bipedicular PVP (PKP). Therefore, the unipedicular procedure is advocated by most surgeons [4–6]. Lateral angulation of the puncture needle is required to approach the anterior third of the vertebral body near midline. As a result, the puncture needle often penetrates the lateral aspect of the pedicle. However, because the usual local anesthetic range is from the skin to the laminar surface, the extrapedicular region is often without anesthesia, with the predictable result of severe pain when the puncture needle passes through this region.

Because of this, it is important to have adequate anesthesia in the extrapedicular region. Accordingly, the authors have added extrapedicular infiltration anesthesia (EPIA) to the conventional local infiltration anesthesia (CLIA) and report the retrospective study of comparing anesthetic effect of CLIA + EPIA with CLIA as a control group for unipedicular PVP (PKP).

## 2. Materials and Methods

### 2.1. General Information

From March 2015 to March 2016, 44 patients (11 males and 33 females) with osteoporotic vertebral compression fractures with a mean age of 71.4 ± 8.8 years (range: 60 to 89) received PVP (PKP). According to the local anesthetic methods, all patients were divided into two groups: 20 patients were managed with CLIA + EPIA and 24 patients with CLIA. Patient perceptions of intraoperative pain were measured with the visual analogue score (VAS). Each patient had brief preoperative teaching to report VAS of 0 as no pain and 10 as maximal pain and to know that they would be asked to report their pain using this scale. Patients were also told that, in the event of severe pain, they could receive additional sedative analgesia. From the time that anesthesia was established to the time that the surgery was completed, patients were asked to report any adverse nerve root effects such as hypesthesia or numbness and any reduction of lower limbs muscle strength. The retrospective study was approved by the ethics committee of the unit and patients provided consent for treatment.

### 2.2. Anesthetic Method

**Figure 1:**
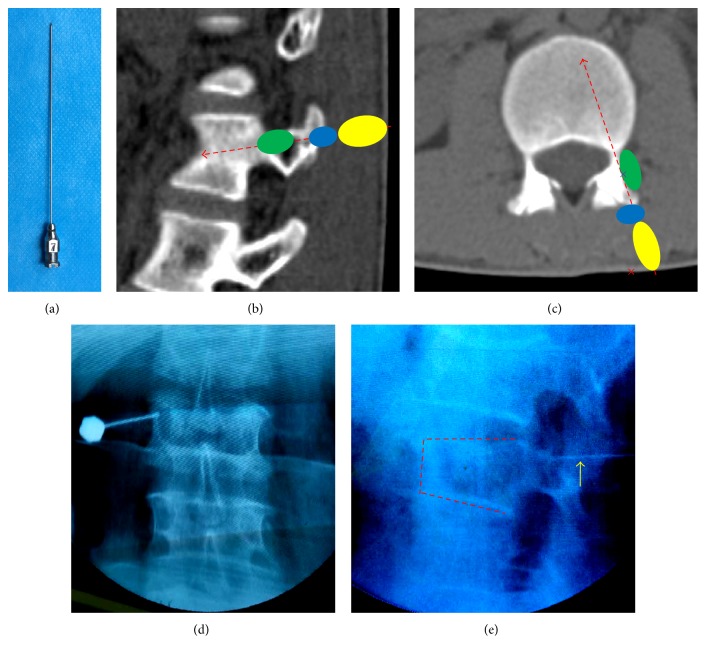
(a) Anesthetic needle (number 7 epidural needle). ((b), (c)) Red dashed arrow-puncture needle path. Yellow area-anesthetic region of the 1st step. Blue area-anesthetic region of the 2nd step. Green area-anesthetic region of the 3rd step (anesthetic region of EPIA). (c) Red ×: pedicle projection point on skin. Purple ×: anesthetic needle position in 3rd step. (d) Anesthetic needle position in 2nd step. (e) Yellow arrow-anesthetic needle position in EPIA on lateral X-ray.

The anesthetic process of CLIA group included two steps. For the first step, the projection point of the pedicle was located, and a number 7 epidural needle (length 9.5 mm, diameter 1 mm) was used for anesthesia; from a point 1 cm lateral to the pedicle projection point, 5 mL of 1% Lidocaine Hydrochloride was used to infiltrate the skin, subcutaneous tissue, and part of the lumbodorsal muscles. In the second step, the anesthetic needle was angled 10–15° with the sagittal plane and was directed toward the laminar periosteum at the pedicular projection point on the lamina where 5 mL of 1% Lidocaine Hydrochloride was injected. Besides the two steps, the anesthetic process of CLIA + EPIA group additionally included the third step, called EPIA. For this step, the anesthetic needle tip first was withdrawn to the subcutaneous tissue. It was then directed toward the lateral half of the pedicle along the lateral superior articular process and the superior border of the transverse process (angulation of 5–10° with the sagittal plane and 5–10° with the coronal plane), and with no withdrawal of blood, 5 mL of 1% Lidocaine Hydrochloride was injected (Figure 1). The total dose of Lidocaine Hydrochloride did not exceed 300 mg, or <4.5 mg/kg.

### 2.3. Statistical Analysis

The age, BMI, and VAS between groups were compared using the unpaired* t*-test, with the level of significance set at *P* < 0.05, using SPSS version 19.

## 3. Results

Patients' demographic characteristics and the results in the two groups were shown in Table 1. There were no significant differences between the two groups in sex, age, and BMI (*P *> 0.05). VAS of CLIA + EPIA group was 2.5 ± 0.7 (range: 1~3), while for the CLIA group, it was 4.3 ± 1.0 (range: 3~7), and there was significant difference (*P* = 0.001) between the two groups. In CLIA + EPIA group, no patients required additional sedative analgesia, but in CLIA group, 1 patient's VAS was 7 and additional sedative analgesia was required. In the two groups, no patients reported nerve root adverse effects.

## 4. Discussion

The anesthetic method for PVP (PKP) usually includes local anesthesia, sedative analgesia, and general anesthesia. General anesthesia has been proven to be effective for surgical procedures and is amenable to intraoperative monitoring and management. However, elderly patients undergoing PVP (PKP) may have poor tolerance of general anesthesia because of frequent concomitant circulatory, respiratory, nervous, and endocrine disorders. Moreover, endotracheal intubation and general anesthesia easily cause respiratory complications, and intraoperative neurological symptoms may not be discovered in a timely manner. Additionally, sedative analgesia with opioids and benzodiazepines carries the potential for respiratory depression, called opioid-induced respiratory depression (OIRD) [7]. Clinical risk factors of OIRD included such comorbid conditions as chronic pulmonary disease, sleep apnea, asthma, chronic kidney, liver impairment, pancreatitis, and traumatic injury [8, 9]. In comparison, the awake surgical patient with local anesthesia is able to express discomfort during the procedure. Surgeons can monitor and adjust the operation process accordingly, and they can also discover neurological symptoms in time to avoid nerve damage. Furthermore, the use of local anesthesia avoids the potential complications of sedative and general anesthesia. But, we must master the dose of the local anesthetic drugs according to patient's BMI, being alert to its toxicity. To sum up, most doctors advocate local anesthesia for PVP (PKP).

Biomechanical and clinical studies have reported that vertebral axial compressive strength would be largely recovered if bone cement is limited to hemivertebra and that bilateral vertebral rigidity would be obtained if bone cement extended past midline [5]. Unipedicular PVP (PKP) can save surgical time, reduce trauma, and reduce surgical complications as compared to the bipedicular procedure. The unilateral procedure does require greater angulation of the needle, which often leads to a vertebral body puncture route that includes the extrapedicular region and periosteum. However, the local anesthetic region usually does not include the extrapedicular periosteum and tissue, and passing the puncture needle pass through this region often results in a severe pain response. Sesay et al. [10] pointed out that, in PVP surgery, pain during needle puncture is more significant than the pain during cutaneous anesthesia and injection of bone cement. The standard of effective local anesthesia is that the verbal rating pain scale (0 = no pain, 1 = mild pain, 2 = moderate pain, and 3 = severe pain) is no more than 2 points [11]. In this study, reported VAS scores during needle puncture in CLIA + EPIA group were less than 3 points, no patients required additional sedative analgesia, and the anesthetic effect was satisfactory.

Usually, local anesthetic for PVP (PKP) ranges only from the skin to the laminar periosteum, essentially only including the first and the second steps of anesthesia. Because of the laminar barrier, the anesthetic is directed mainly toward the outer surfaces of the lamina and paraspinal muscles, and the extrapedicular periosteum and deeper extrapedicular soft tissue are not anesthetized. However, the acute angulation of the puncture needle will result in penetration of the extrapedicular region, which may result in severe pain. EPIA can anesthetize the entire extrapedicular periosteum and soft tissues by directing the anesthetic needle to the lateral 1/2 of the pedicle and infusing local anesthetic. To avoid nerve root anesthesia and lumbar artery damage, the needle should not enter too deeply into the junction of pedicle and vertebral body. C-arm X-ray monitoring can aid with precise localization of the anesthetic needle. In the event of articular process hyperplasia at lower lumbar levels, angulation of the anesthetic needle could be increased to 10–15°. At thoracic vertebral levels, it is safe to contact the ribs, but care should be taken to avoid piercing the pleura.

## 5. Conclusion

EPIA deserves clinical consideration for unipedicular PVP (PKP), as it provides effective anesthesia and presents no significant complications. However, it may slightly increase X-ray radiation exposure for precise localization of the anesthetic needle.

## Figures and Tables

**Table 1:** Patients' demographic characteristics and the results in the two groups.

	CLIA + EPIA group	CLIA group	*P* value
Number of cases	20	24	
Sex, male : female	4 : 16	7 : 17	0.484^†^
Age (yrs)	72.4 ± 9.5 (62~89)	70.5 ± 8.2 (60~87)	0.287
Body mass index (BMI) (kg/m^2^)	22.9 ± 2.7 (18.8~28.3)	22.6 ± 2.8 (17.3~28.9)	0.785
Injured vertebral			
T12	5	9	
L1	5	7	
L2	4	5	
L3	6	1	
L4	0	2	
VAS	2.5 ± 0.7	4.3 ± 1.0	0.001
Additional sedative analgesia	0	1	
Nerve root adverse effects	0	0	

^†^Chi-square test.

## Abbreviations

EPIA: Extrapedicular infiltration anesthesia
CLIA: Conventional local infiltration anesthesia
PVP: Percutaneous vertebroplasty
PKP: Percutaneous kyphoplasty
VAS: Visual analogue score.
